# Introductory Lecture before the Albany Medical College, Delivered October 1, 1839

**Published:** 1839-11-02

**Authors:** 


### BIBLIOGRAPHICAL NOTICE.

*Introductory Lecture before the Albany Medical College, delivered October* 1, 1839. By Gunning S. Bedford, M. D., Professor of Obstetrical Medicine. Albany: 1839.

The season for the *introductories* has again come round; and, from the number of professors who make their début this year, we may anticipate a liberal supply of these publications. Coming, as they generally do, from able writers, we often gather from them useful information and sound views upon the important subject of medical education. The lecture of Professor Bedford is of this character. He has entered upon the topic of collegiate endowments, and makes an able appeal in behalf of the equal claims of medicine with other branches of learning. This appeal is responded to in Albany. The Medical Faculty of the Albany College has received the very handsome endowment of a building, fixtures, and scientific apparatus. It of course stands upon decided vantage ground, and the professors are very naturally sanguine of success. From their known talent, their advantages of geographical situation, as well as from their unencumbered pecuniary position, it is quite probable that these expectations will be realized.

